# Effects of Drying Methods on the Phytochemical Contents, Antioxidant Properties, and Anti-Diabetic Activity of *Nasturtium officinale* R.Br. (Betong Watercress) from Southern Thailand

**DOI:** 10.3390/life14091204

**Published:** 2024-09-23

**Authors:** Praporn Kijkuokool, Irina Stepanov, Sakaewan Ounjaijean, Pimpisid Koonyosying, Kittipan Rerkasem, Hataichanok Chuljerm, Wason Parklak, Kanokwan Kulprachakarn

**Affiliations:** 1 School of Health Sciences Research, Research Institute for Health Sciences, Chiang Mai University, Chiang Mai 50200, Thailand; praporn_k@cmu.ac.th (P.K.); sakaewan.o@cmu.ac.th (S.O.); rerkase@gmail.com (K.R.); hataichanok.ch@cmu.ac.th (H.C.); wason.p@cmu.ac.th (W.P.); 2 Division of Environmental Health Sciences, School of Public Health and Masonic Cancer Center, University of Minnesota, Minneapolis, MN 55455, USA; stepa011@umn.edu; 3 Department of Biochemistry, Faculty of Medicine, Chiang Mai University, Chiang Mai 50200, Thailand; pimpisid.k@cmu.ac.th; 4 Department of Surgery, Faculty of Medicine, Chiang Mai University, Chiang Mai 50200, Thailand

**Keywords:** *Nasturtium officinale* R.Br., Betong watercress, drying method, total glucosinolate content, polyphenol content, antioxidant activity, anti-diabetic activity

## Abstract

*Nasturtium officinale* R.Br. (Betong watercress) contains high levels of secondary metabolites that offer health benefits. However, fresh-cut watercress has a short shelf life. This study aimed to assess the effect of drying methods on the phytochemical contents, antioxidant activity, and anti-diabetic activity of Betong watercress. The watercress was dried using three methods: roasting (R, 50 °C, 40 min); roasting and drying (RD, 40 min roasting at 50 °C and 1 h drying at 80 °C); and blanching, roasting, and drying (BRD, 30 s blanching at 80 °C, 20 min roasting at 50 °C, and 1 h drying at 80 °C). Aqueous extracts from each drying method were analyzed for total phenolic content, total flavonoid content, total glucosinolate content, antioxidant activities (FRAP, DPPH, and ABTS assays), and α-amylase enzyme inhibition. From the results, the R method provided the highest level of total phenolic, total flavonoid, and total glucosionolate content compared to the RD and BRD methods. Similarly, antioxidant activities and α-amylase enzyme inhibition were highest in the R method, followed by the RD and BRD methods. Our results demonstrate that roasting of Betong watercress without the addition of blanching or drying effectively preserves the phytochemical contents, antioxidant activities, and anti-diabetic activity.

## 1. Introduction

Plants have been closely linked to human life and have been used in traditional therapies for millennia. According to the World Health Organization (WHO), medicinal plants and their derivatives serve as the basic treatment for up to 80% of the global population [1]. *Nasturtium officinale* R.Br., commonly known as watercress, is an aquatic leafy cruciferous vegetable belonging to the Brassicaceae family [2] and is native to Asia, Europe, India, and Africa [3,4]. The European Food Safety Authority recognizes watercress as a safe edible vegetable among herbs, edible flowers, and leafy vegetables [5]. Further, the United States Centers for Disease Control and Prevention have identified watercress as one of the vegetables with the highest nutrient density relative to its caloric content [6]. Watercress is rich in vitamins A, C, E, and B3, as well as essential minerals such as phosphorus, calcium, iron, iodine, manganese, and copper. It also contains abundant bioactive compounds, including glucosinolates and their derivatives; isothiocyanates, which are the breakdown products of glucosinolates; and polyphenolic compounds like quercetin, rutin, kaempferol, coumaric acid, and ferulic acid [7,8,9,10,11]. These diverse secondary metabolites are likely to be responsible for the significant health benefits of watercress consumption and supplementation, as demonstrated in numerous in vitro and in vivo studies. These benefits include glucose and lipid modulation, antioxidant system enhancement, anti-inflammatory effects, nephroprotective and hepatoprotective properties, DNA damage mitigation, anticancer activity, and anti-tuberculosis potential [4,12,13,14,15].

Initially brought into Thailand by immigrants from China, watercress is now extensively cultivated in the southern region of Thailand, where it is locally known as “Phak Nam Betong” or “Betong watercress” [16]. This offers opportunities for research to better characterize the potential of watercress to improve public health in Thailand and for consumer communication to promote the consumption of watercress. However, a key challenge is that watercress is highly perishable due to its aquatic nature, with a shelf life of approximately seven days. Additionally, factors such as harvesting procedures, storage conditions, transportation, and processing operations affect the shelf life and quality of watercress as well [17]. Indeed, watercress is typically consumed as a fresh-cut vegetable and used in salads [18].

Several methods can be used for extending the shelf life of plant products. For example, drying extends storage time by transferring water molecules from the plant matrix to the surrounding environment, thereby enhancing the microbiological stability of the plant [19]. Various techniques are used for drying, with the predominant ones being conduction drying, which directly transfers heat to the plant product (e.g., roasting); convection, which transfers heat through hot gases to the plant product (e.g., using hot air or oven drying); and radiation- or sun-drying [20]. Pretreatment methods such as blanching, boiling water, osmotic dehydration, vacuum, ultrasound, and electric fields can further enhance the drying process by increasing the drying rate, improving moisture removal efficiency, and helping retain color and sensory attributes [21].

Some studies explored the impact of such processing methods on Betong watercress; however, studies are limited and used different outcome measures. For example, Tayeh [22] developed a Betong watercress tea using different drying procedures, including roasting and drying, and used sensory evaluation score as the outcome. Aksornthong et al. [23] studied the effects of cooking in the microwave at 800 W for 3 min on the antitumor effect of Betong watercress in rats. That study measured levels of phenethyl isothiocyanate (PEITC), a key chemopreventive agent in watercress, and showed that cooked watercress (5.56 g/mg dry powder) contained a higher level of PEITC than fresh watercress (1.46 g/mg dry powder). However, rats fed cooked watercress exhibited significantly lower CYP1A2 metabolic activity, as measured by caffeine metabolic ratios, compared to rats given fresh watercress.

Given the potential of Betong watercress as a natural health-protective dietary component in Thailand and its perishable nature, systematic research on the effects of processing methods on its phytochemical content and biological potential is needed. Our study aimed to address this gap by comparing the effects of various drying methods, including combinations of roasting (conduction drying) and hot air drying (convection drying), with the pretreatment method (blanching), on the total glucosinolate content, polyphenol contents, antioxidant activities, and the ability to inhibit the α-amylase enzyme.

## 2. Materials and Methods

### 2.1. Plant Material and Drying Processes

**Figure 1:**
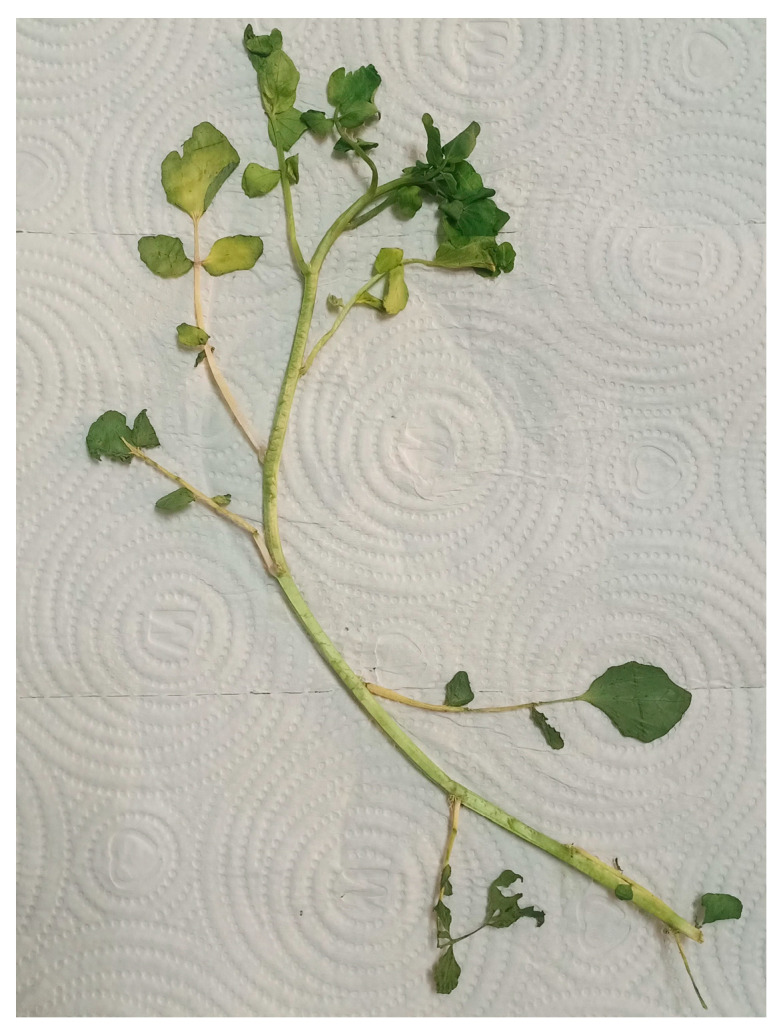
A voucher specimen of Betong watercress (*Nasturtium officinale* R.Br.) collected from the Zhi Wu watercress farm, Betong district, Yala province, Thailand.

*Nasturtium officinale* R.Br. was obtained in April 2023 from the Zhi Wu watercress farm, an organic farm located in Betong, Yala, Southern Thailand. The plants were harvested approximately six weeks after planting. The authenticity of Betong watercress was verified (No. 0023371) (Figure 1) by the Department of Pharmaceutical Sciences, Faculty of Pharmacy, Chiang Mai University. Fresh Betong watercress was cleaned and desiccated. The drying processes were modified from the protocol described by Rajchasom et al. [24].

- Roasting (R): Betong watercress was roasted in a cooking pan at 50 °C for 40 min.
- Roasting and drying (RD): Betong watercress was roasted in a cooking pan at 50 °C for 20 min. It was then dried in an electric hot air dryer (Model ET450-16T, Yok Intertrade, Chiang Mai, Thailand) at 80 °C for one hour.
- Blanching, roasting, and drying (BRD): Betong watercress was blanched at 80 °C for 30 s and rapidly cooled in cold water. It was then desiccated until almost dry, roasted in a cooking pan at 50 °C for 20 min, and finally dried in an electric hot air dryer at 80 °C for one hour.

The weight of Betong watercress was recorded before and after the drying process to determine the drying yield, which was calculated using Equation (1):

Drying yield (%) = (M_dw_/M_fw_) × 100 (1)

where M_dw_ represents the weight after the drying process, and M_fw_ represents the fresh weight of the plant. Each sample was ground into a fine powder using an electric grinder (Jing Gong Yi, Jakarta Barat, Indonesia). The Betong watercress powder was stored in a portable desiccator (Model Auto C-3W, Sanplatec, Osaka, Japan) and protected from light until further analysis.

### 2.2. Preparation of Watercress Aqueous Extract

The preparation the aqueous extract was adapted from was adapted from the method described by Doheny-Adams et al. [25]. A total of 10 g of dried Betong watercress powder was boiled in 100 mL of hot water at 80 °C for 20 min, maintaining a 1:10 ratio. After cooling, the extract was filtered through Whatman No. 1 filter paper using suction filtration. The filtrate was then lyophilized using a freeze dryer (Model Alpha 1–4 LSCplus, Martin Christ, Osterode am Harz, Germany) and stored at −20 °C until further use. The weight of the plant extract was recorded, and the extraction yield was calculated using Equation (2):

Extraction yield (%) = (M_ew_/M_dw_) × 100 (2)

where M_ew_ is the weight of the lyophilized plant extract, and M_dw_ is the weight of the dried plant powder.

### 2.3. Total Glucosinolate Content

The total glucosinolate content was determined using the method described by Ishida et al. [26]. In this procedure, 13.3 µL of each Betong watercress extract was mixed with 20 µL of ultrapure water and 200 µL of 2 mM palladium (II) chloride reagent (PdCl_2_), prepared by dissolving 35.5 mg of PdCl_2_ (Sigma-Aldrich, Darmstadt, Germany) in 168 µL of concentrated hydrochloric acid (conc. HCl) and diluting the solution to 100 mL with ultrapure water. The mixture was incubated at room temperature for 1 h. The glucosinolate in the sample reacts with palladium chloride, causing a color change from light brown to dark brown. The absorbance was measured at 425 nm using a spectrophotometer (BMG LABTECH, Ortenberg, Germany). The absorbances of the blank, using ultrapure water instead of the sample, and pair-blank, using ultrapure water instead of the palladium chloride, were subtracted from the sample absorbance. Total glucosinolate content was calculated using a standard calibration curve of sinigrin (Sigma-Aldrich, Darmstadt, Germany) (0.05, 0.1, 0.25, 0.75, 1, 2 mg/mL) and expressed as mg sinigrin equivalent (SE) per gram of extract. The experiment was carried out in triplicate.

### 2.4. Total Phenolic Content

The total phenolic content was determined using the Folin–Ciocalteu (FC) colorimetric method, as modified by Uyumlu and Çağlar Yılmaz [27]. The FC reagent (Sigma-Aldrich, Darmstadt, Germany), which contains heteropoly acid (phosphomolybdate-phosphotungstate), oxidizes the phenol or phenolic-hydroxy groups in the samples. The reaction leads to the formation of a molybdenum–tungsten complex, which exhibited a blue color [28]. The FC working reagent was prepared by diluting the FC reagent with deionized water at a 1:10 ratio. In a 96-well plate, 20 µL aliquots of each Betong watercress extract were mixed with 100 µL of FC working reagent. The mixture was incubated for 3 min before adding 80 µL of 1 M sodium carbonate (Na_2_CO_3_) (Sigma-Aldrich, Darmstadt, Germany) solution. The absorbance was measured at 765 nm using a spectrophotometer. A standard calibration curve of gallic acid (ranging from 0 to 0.5 mg/mL) (Sigma-Aldrich, Darmstadt, Germany) was used to quantify the total phenolic content, expressed as mg gallic acid equivalent (GAE) per gram of extract. The experiment was conducted in triplicate.

### 2.5. Total Flavonoid Content

The total flavonoid content in Betong watercress extracts was determined using a method modified from Aryal et al. [29]. In a 96-well plate, 50 µL of each Betong watercress extract was mixed with 10 µL of 10% aluminum chloride (AlCl_3_) (Sigma-Aldrich, Darmstadt, Germany), 10 µL of 1 M sodium acetate (CH_3_COONa) (Sigma-Aldrich, Darmstadt, Germany), and 150 µL of 50% methanol. The mixture was equilibrated at 25 °C for 30 min. The absorbance was then measured at 415 nm using a spectrophotometer. The optical density (OD) of the blank was measured and subtracted from the OD of the sample. The total flavonoid content was calculated using a standard calibration curve of quercetin (Sigma-Aldrich, Darmstadt, Germany) at concentrations ranging from 0 to 0.5 mg/mL and expressed as mg quercetin equivalent (QE) per gram of extract. The experiment was carried out in triplicate.

### 2.6. Antioxidant Activities

The antioxidant power of Betong watercress extracts was assessed using a ferric reducing antioxidant power (FRAP) assay. Additionally, the samples’ ability to scavenge free radicals was evaluated using the 2,2-diphenyl-1-picrylhydrazyl hydrate (DPPH) radical scavenging assay and the 2,2′-azino-bis (3-ethylbenzthiazoline-6-sulphonic acid) (ABTS) radical scavenging assay.

#### 2.6.1. Assay of Ferric Reducing Antioxidant Power (FRAP)

The FRAP assay was conducted following the method described by Taşkın et al. [30], with slight modifications. The FRAP working solution was prepared by mixing 10 mL of acetate buffer (300 mM, pH 3.6) (Sigma-Aldrich, Darmstadt, Germany), 1 mL of ferric chloride hexahydrate (FeCl_3_•6H_2_O, 20 mM) (Sigma-Aldrich, Darmstadt, Germany), and 1 mL of 2,4,6-tripyridyl-s-triazine (TPTZ, 10 mM) (Sigma-Aldrich, Darmstadt, Germany). In a 96-well plate, 5 µL of each Betong watercress extract was combined with 180 µL of FRAP working solution and incubated at 37 °C for 5 min. The absorbance was then measured at 593 nm using a spectrophotometer. The antioxidant activity was measured based on the reduction of ferric-2,4,6-tripyridyl-s-triazine (Fe^3+^-TPTZ) to ferrous-2,4,6-tripyridyl-s-triazine (Fe^2+^-TPTZ), forming a blue complex [31]. A standard calibration curve for trolox (Sigma-Aldrich, Darmstadt, Germany) was set at concentrations ranging from 0 to 1 mg/mL. The antioxidant activity, expressed as FRAP value, was calculated and reported as mg trolox equivalents (TE) per gram of extract. The assay was conducted in triplicate.

#### 2.6.2. Assay of DPPH Radical Scavenging Activity

The DPPH radical scavenging activity of Betong watercress extracts was evaluated using a method based on Aires et al. [32], with some modifications. The DPPH radical solution was prepared by dissolving 1.18 mg of DPPH (Sigma-Aldrich, Darmstadt, Germany) in 25 mL of 95% ethanol. The assay was performed by combining 10 µL of each Betong watercress extract at different concentrations (0.5, 1, 2.5, 5, 10, 20 mg/mL) with 195 µL of DPPH radical solution in a 96-well plate. The mixture was incubated in the dark at 25 °C for 30 min. Subsequently, the absorbance was measured at 517 nm using a spectrophotometer. Antioxidants in the sample donate a hydrogen atom, reducing the violet-colored DPPH radical to the colorless or yellow 2,2-diphenyl-1-hydrazine (DPPH-H) [33]. A standard calibration curve of trolox at concentrations ranging from 0 to 0.2 mg/mL was used to quantify the antioxidant activity, expressed as the DPPH radical scavenging value, in mg TE per gram of extract. The OD of the sample was calculated against the OD of the blank using the following formula (Equation (3)) to determine the scavenging activity, or the DPPH radical percentage of inhibition:

DPPH radical scavenging activity (%) = [(OD_blank_ − OD_sample_)/OD_blank_] × 100 (3)

The inhibition percentage was used to determine the IC50 value, the concentration required to inhibit 50% of free radicals. The assay was conducted in triplicate.

#### 2.6.3. Assay of ABTS Radical Scavenging Activity

The ABTS radical scavenging assay was carried out following a method adapted from Arulvendhan et al. [34]. The ABTS radical solution was prepared by mixing 7 mM ABTS (Sigma-Aldrich, Darmstadt, Germany) with 2.45 mM potassium persulfate (K_2_S_2_O_8_) (Sigma-Aldrich, Darmstadt, Germany) in an equal ratio. The ABTS radical solution was equilibrated at room temperature for 12 to 16 h in the dark. Before use, the ABTS solution was adjusted to an absorbance of 0.7 ± 0.02 at 743 nm using ethanol. In this assay, 10 µL of each Betong watercress extract (0.5, 1, 2.5, 5, 10, 20 mg/mL) was combined with 195 µL of the ABTS radical solution and incubated for 30 min at room temperature, away from sunlight. The absorbance was then measured at 734 nm using a spectrophotometer. Antioxidants in the sample reduce the ABTS radical, resulting in decolorization [35]. The antioxidant activity of Betong watercress extracts was calculated from the standard calibration curve of trolox at concentrations ranging from 0 to 0.2 mg/mL. It was reported as the ABTS radical scavenging value in mg TE per gram of extract. The ABTS radical scavenging percentage was calculated using Equation (4):

ABTS radical scavenging activity (%) = [(OD_blank_ − OD_sample_)/OD_blank_] × 100 (4)

The inhibition percentage was used to determine the ABTS IC50 value for each extract. The experiment was conducted in triplicate.

### 2.7. Assay of Pancreatic α-Amylase Inhibition

Pancreatic α-amylase inhibition activity was measured using a modified starch-iodide method from Ononamadu et al. [36]. The assay was performed with Betong watercress extracts at a concentration of 1 mg/mL. Phosphate buffer (0.1 M, pH 6.9) was used as the blank, and acarbose (0.5–8 mM) (Sigma-Aldrich, Darmstadt, Germany) was used as the standard solution. In a 96-well plate, 50 µL of the sample was mixed with 50 µL of the starch solution (3 mg/mL). The mixture was incubated at 37 °C for 5 min, followed by the addition of 50 µL of the α-amylase enzyme solution (20 units/mL) (Sigma-Aldrich, Darmstadt, Germany). The mixture was re-incubated at 37 °C for 15 min. The reaction was stopped by adding 50 µL of hydrochloric acid solution (HCl, 1 M) and 50 µL of 1% iodine solution (Sigma-Aldrich, Darmstadt, Germany). The absorbance measurement was performed at 650 nm using a spectrophotometer. The test was performed in triplicate. The percentage of enzyme inhibition was calculated using Equation (5):

% Inhibition = [1 − [(ODblank − ODsample)/ODblank]] × 100(5)

### 2.8. Statistical Analysis

All statistical analyses and graphical presentations were performed using GraphPad Prism Software version 10.0 for Windows. Experiments were conducted in triplicate, and results are reported as the mean ± standard deviation (SD). Data normality was assessed using the Shapiro–Wilk test. One-way analysis of variance (ANOVA) was used to compare the differences between drying methods, followed by Tukey’s post hoc test. A *p*-value of less than 0.05 was considered statistically significant.

## 3. Results

### 3.1. Determination of Drying Yields and Extraction Yields of Betong Watercress Extracts

**Figure 2:**
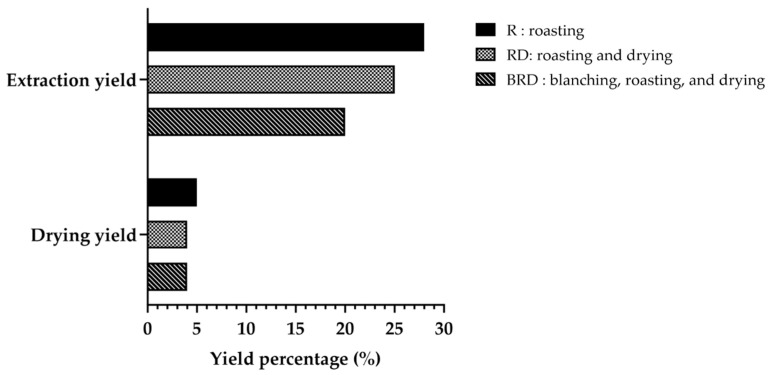
Drying yields and extraction yields of Betong watercress with different drying processes, expressed as yield percentage (%).

The results for drying yields and extraction yields of Betong watercress are presented in Figure 2. After drying, Betong watercress dried by the roasting (R) method showed the greatest yield (5.51%); followed by the roasting and drying (RD) method and the blanching, roasting, and drying (BRD) method (4.82% and 4.41%, respectively). The extraction yields followed a similar trend, with the R method showing the highest yield, followed by the RD method and the BRD method (28.58%, 25.90%, and 20.09%, respectively).

### 3.2. Determination of Total Phenolic, Total Flavonoid, and Total Glucosinolate Contents of Betong Watercress Extracts

The results for the polyphenol contents of Betong watercress, including the total phenolic content and the total flavonoid content, are shown in Table 1. The total phenolic content was significantly higher (*p* < 0.05) in the R method (36.27 ± 2.99 mg GAE/g extract) compared to the RD method (28.72 ± 1.21 mg GAE/g extract) and the BRD method (24.46 ± 1.56 mg GAE/g extract). There was no statistically significant difference (*p* > 0.05) in total phenolic content between the RD and BRD methods.

The R method also showed the highest total flavonoid content (6.58 ± 0.65 mg QE/g extract), which was significantly higher than (*p* < 0.05) the BRD method (4.38 ± 0.60 mg QE/g extract). The flavonoid content of the RD method (5.57 ± 0.08 mg QE/g extract) was in between the other two methods, but there was no significant difference (*p* > 0.05).

**Figure 3:**
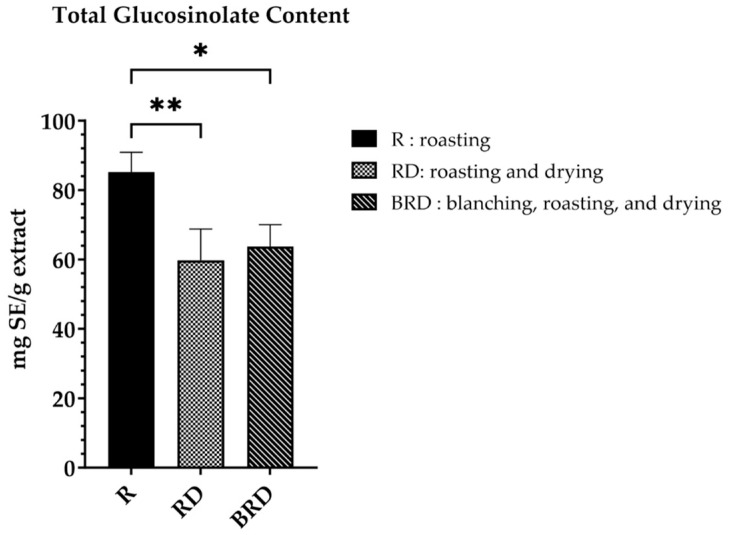
Total glucosinolate content of Betong watercress extracts with different drying processes, expressed as mg sinigrin equivalent (SE) per gram extract. Data are mean ± SD of triplicate determinations. * Significantly different (*p* < 0.05). ** Significantly different (*p* < 0.01). SD, standard deviation.

The total glucosinolate content of Betong watercress extracts is presented as a bar graph (Figure 3). The R method exhibited significantly higher (*p* < 0.05) glucosinolate content (85.16 ± 5.70 mg SE/g extract) than the RD and BRD methods (59.78 ± 9.00 and 63.78 ± 6.24 mg SE/g extract, respectively). Similar to the total phenolic content, there was no significant difference (*p* > 0.05) in the total glucosinolate content between the RD and BRD methods.

### 3.3. Antioxidant Activities of Betong Watercress Extracts

**Figure 4:**
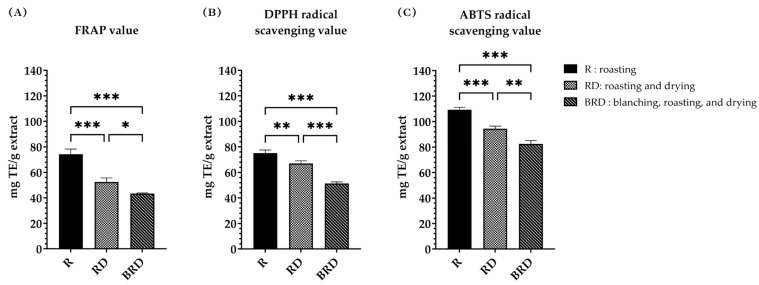
Antioxidant activities of Betong watercress extracts with different drying processes. (**A**) FRAP value; (**B**) DPPH radical scavenging value; and (**C**) ABTS radical scavenging value were expressed as mg trolox equivalent (TE) per gram extract. Data are mean ± SD of triplicate determinations. * Significantly different (*p* < 0.05). ** Significantly different (*p* < 0.01). *** Significantly different (*p* < 0.001). FRAP, ferric reducing antioxidant power; DPPH, 2,2-diphenyl-1-picrylhydrazyl hydrate; ABTS, 2,2’-azino-bis (3-ethylbenzthiazoline-6-sulphonic acid) radical scavenging; SD, standard deviation.

**Figure 5:**
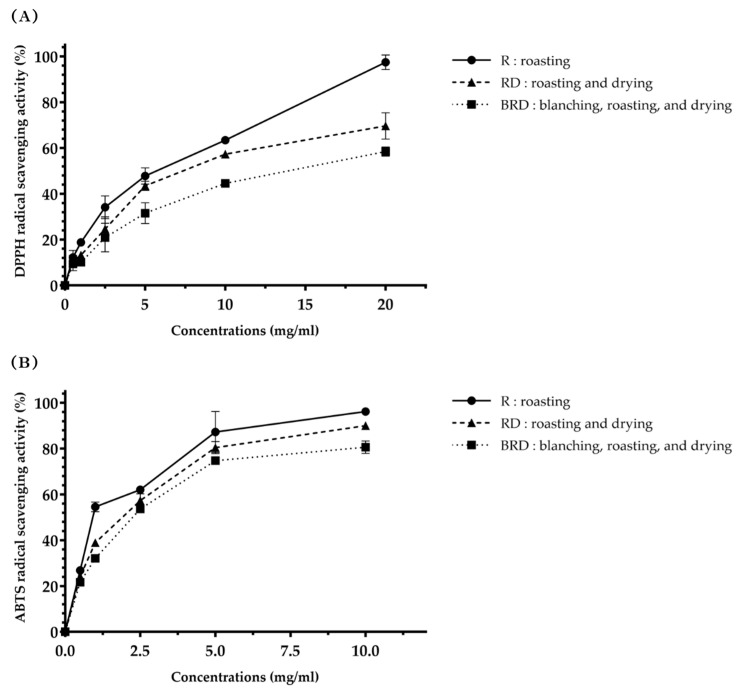
Free radical scavenging activities (%) of Betong watercress extracts with different drying processes at different concentrations. (**A**) DPPH radical scavenging percentage and (**B**) ABTS radical scavenging percentage. Data are mean ± SD of triplicate determinations. DPPH, 2,2-diphenyl-1-picrylhydrazyl hydrate; ABTS, 2,2’-azino-bis (3-ethylbenzthiazoline-6-sulphonic acid) radical scavenging; SD, standard deviation.

**Figure 6:**
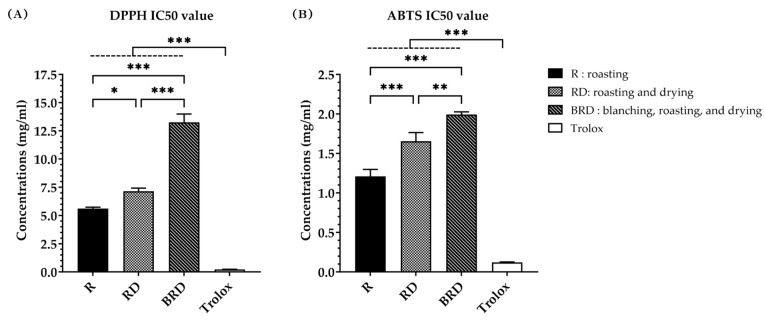
IC50 value of trolox and Betong watercress extracts with different drying processes. (**A**) DPPH IC50 value and (**B**) ABTS IC50 value were expressed as mg/mL. Data are mean ± SD of triplicate determinations. * Significantly different (*p* < 0.05). ** Significantly different (*p* < 0.01). *** Significantly different (*p* < 0.001). DPPH, 2,2-diphenyl-1-picrylhydrazyl hydrate; ABTS, 2,2’-azino-bis (3-ethylbenzthiazoline-6-sulphonic acid) radical scavenging; SD, standard deviation.

The results of the measurements of antioxidant activities of Betong watercress extracts using FRAP, DPPH radical scavenging, and ABTS radical scavenging assays are illustrated in Figure 4, Figure 5 and Figure 6.

In the FRAP assay results, shown in Figure 4A, there was a significant difference (*p* < 0.05) in FRAP values among the drying methods. The FRAP value refers to the potential of the samples to reduce the Fe^3+^-TPTZ complex. The R method exhibited the highest FRAP value (74.18 ± 4.09 mg TE/g extract), followed by the RD method (52.51 ± 3.12 mg TE/g extract) and the BRD method (43.42 ± 0.35 mg TE/g extract).

The antioxidant activity from the DPPH radical scavenging assay was expressed as the DPPH radical scavenging value (mg TE/g extract), the DPPH radical scavenging percentage (%), and the DPPH IC50 value. The DPPH radical scavenging value of Betong watercress extracts is shown in Figure 4B. The R method demonstrated a significantly higher (*p* < 0.01) DPPH radical scavenging value (75.06 ± 2.45 mg TE/g extract) compared to the RD and BRD methods (66.99 ± 2.06 and 51.40 ± 1.20 mg TE/g extract, respectively). The RD method also had a significantly higher (*p* < 0.001) DPPH radical scavenging value than the BRD method. Figure 5A presents the DPPH radical scavenging percentage. At the same concentration, the R method showed the highest ability to scavenge DPPH radicals. The DPPH radical scavenging percentage of each extract was then used to calculate the IC50 value, which represents the concentration needed to scavenge 50% of DPPH radicals. The comparison of the DPPH IC50 values of three extracts to trolox (Figure 6A) showed that trolox had the lowest DPPH IC50 value (0.22 ± 0.01 mg/mL), significantly different (*p* < 0.001) from the watercress extracts. There were statistically significant differences (*p* < 0.05) in the DPPH IC50 values among watercress extracts. The R method had the lowest DPPH IC50 value (5.61 ± 0.12 mg/mL), followed by the RD and BRD methods (7.15 ± 0.26 and 13.25 ± 0.74 mg/mL, respectively).

The ABTS radical scavenging assay findings were expressed as the ABTS radical scavenging value (mg TE/g extract), the ABTS radical scavenging percentage (%), and the ABTS IC50 value. The ABTS radical scavenging values of Betong watercress extracts showed a similar trend to the DPPH radical scavenging values (Figure 4C). All drying methods showed statistically significant differences (*p* < 0.01) in ATBS radical scavenging values. The R method had the greatest ABTS radical scavenging value (109.20 ± 1.99 mg TE/g extract), followed by the RD method (94.36 ± 2.10 mg TE/g extract) and the BRD method (82.37 ± 2.73 mg TE/g extract). Figure 5B displays the ABTS radical scavenging percentage. At the same concentration, the R method extract exhibited a higher ABTS radical inhibition percentage compared to the RD and BRD methods. The ABTS IC50 values, shown in Figure 6B, reveal that trolox had the lowest ABTS IC50 value (0.12 ± 0.00 mg/mL), which was significantly lower than the ABTS IC50 values of all watercress extracts (*p* < 0.001). Among the watercress extracts, the R method exhibited the lowest ABTS IC50 values (1.21 ± 0.09 mg/mL), followed by the RD method (1.65 ± 0.11 mg/mL) and the BRD method (1.99 ± 0.03 mg/mL), respectively. These differences in ABTS IC50 values were statistically significant (*p* < 0.01).

### 3.4. α-Amylase Enzyme Inhibition of Betong Watercress Extracts

**Figure 7:**
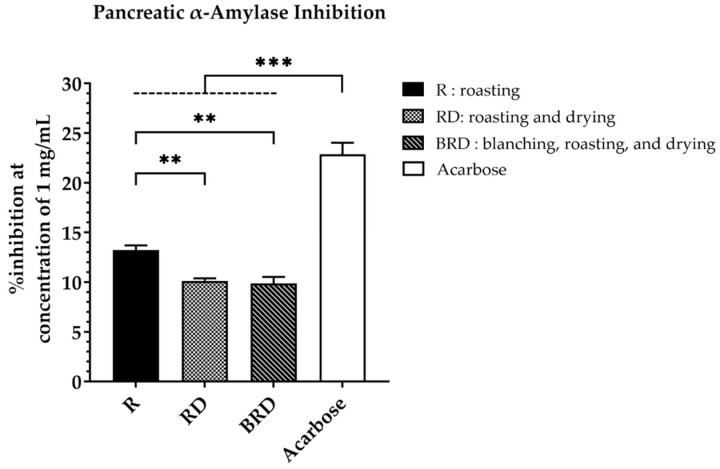
Pancreatic α-amylase inhibition activity (%) of acarbose and Betong watercress extracts with different drying processes at a concentration of 1 mg/mL. Data are mean ± SD of triplicate determinations. ** Significantly different (*p* < 0.01). *** Significantly different (*p* < 0.001). SD, standard deviation.

The anti-diabetic potential of Betong watercress extracts was evaluated by measuring their ability to inhibit the pancreatic α-amylase enzyme. Figure 7 shows the enzyme inhibition percentages of Betong watercress extracts and acarbose at a dosage of 1 mg/mL. Among Betong watercress extracts, the R method showed the greatest inhibition percentage (13.21 ± 0.48%), which was significantly greater than (*p* < 0.01) the RD method (10.12 ± 0.27%) and the BRD method (9.89 ± 0.62%). No significant difference was found between the RD and BRD methods (*p* > 0.05). However, all watercress extracts showed significantly lower inhibition percentages compared to acarbose (22.87 ± 1.15%) (*p* < 0.001).

## 4. Discussion

This paper adds to the literature on watercress by analyzing the phytochemicals and antioxidant properties of the Betong watercress, a specific variety grown and consumed in Thailand, and by assessing the effects of drying techniques on such properties. The phytochemical analysis indicates that Betong watercress is rich in secondary metabolites. Our results demonstrated high levels of polyphenols, including total phenolic content (24.46 to 36.27 mg GAE/g extract) and total flavonoid content (4.38 to 6.58 mg QE/g extract). Among the drying methods that could be used to prolong the use of the otherwise perishable watercress, the roasting method resulted in the highest levels of total phenolic and flavonoid contents. Additional steps in the processing, such as drying and blanching, resulted in reductions of the contents of these beneficial compounds.

Our results align with previous studies that highlight the effectiveness of roasting in enhancing phenolic content. For example, Kittibunchakul et al. [37] investigated the drying of Sacha inchi seeds using various methods, including roasting in a cooking pan, drying in a vacuum oven, and hot air drying. The results demonstrated that the roasting method showed the highest total phenolic content (58.13 mg GAE/100 g dried weight), significantly greater than the phenolic content of the hot air drying method (40.83 mg GAE/100 g dried weight). Interestingly, the roasted seeds even had a higher total phenolic content than the raw seeds (45.15 mg GAE/100 g dried weight). This suggests that roasting is particularly effective at liberating phenolic compounds.

Phenolic compounds in plants exist in various forms, including free form; esterified form; and insoluble-bound form, which is the predominant form. The majority of insoluble-bound phenolics, such as phenolic acids and flavonoids, are found in the cell wall matrix of plants. They bond to cell wall substances such as pectin, cellulose, arabionoxylan, and structural proteins via covalent bonds. Thermal treatment, particularly roasting, can disrupt the cell wall matrix and then liberate these bound phenolic compounds [38]. Additionally, roasting can promote the formation of heat-induced extractable polyphenols, resulting in higher levels of total phenolic and flavonoid contents in the extracts [39].

In contrast, other drying methods, such as hot air drying, yield different outcomes. According to An et al. [40], hot air drying reduced the total phenolic content in ginger from 11.97 to 9.69 mg GAE/g dried weight compared to the fresh plant. Similarly, the total flavonoid content decreased from 13.49 to 12.08 mg GAE/g dried weight. These results align with our finding that combining drying methods leads to a reduction in the phytochemical contents of Betong watercress.

While the literature indicates some advantages of combining drying methods with pretreatments, hydrothermal pretreatments, particularly blanching and boiling, may reduce secondary metabolite levels. Thuwapanichayanan et al. [41] demonstrated that the drying method provided a significantly higher phenolic content in ginger (18.62 to 19.00 mg GAE/g dried weight) compared to raw ginger (15.41 mg GAE/g dried weight). However, when blanching was added as a pretreatment, the phenolic content decreased (16.90 to 17.76 mg GAE/g dried weight). The reduction in phenolic compounds during hydrothermal processes can be attributed to two main factors. First, some phenolic compounds may leach into the blanching water [42]. Second, phenolic compounds may form irreversible covalent bonds during the hydrothermal treatment. Therefore, these phenolic compounds may not be hydrolyzed and extracted efficiently during the extraction process [38].

Regarding the glucosinolate content in Betong watercress, our findings indicate a high level of glucosinolates. Among the drying methods, roasting alone proved to be the most effective at preserving glucosinolate content, while combining roasting with drying or blanching resulted in decreased glucosinolate levels.

Aires et al. [43] studied the effects of various cooking methods on the glucosinolate content of broccoli and white cabbage, both members of the Brassica family. They found that boiling significantly reduced the glucosinolate content in fresh broccoli from 30.87 to 16.07 µmol/g dried weight compared to microwave and steaming methods. Similarly, for white cabbage, boiling caused the greatest loss in glucosinolate content, decreasing from 19.25 to 14.81 µmol/g dried weight. These results support our findings, as the inclusion of hydrothermal pretreatment (blanching) in our methods led to a reduction in glucosinolate levels.

The reduction of glucosinolate content during thermal processing can be explained by several mechanisms. Generally, cruciferous plants contain an enzyme called β-thioglucosidase, or mirosinase. This enzyme is activated when plant cells are mechanically disrupted, such as chewing, chopping, or cutting. Once activated, myrosinase catalyzes the conversion of glucosinolates into various biologically beneficial by-products such as phenethyl isothiocyanate, isothiocyanates, thiocyanates, and nitriles [44]. However, severe heat treatments, such as high-temperature treatments or multiple thermal processes, can degrade both glucosinolates and myrosinase enzymes. According to a review by Lafarga et al. [45], thermal treatments with temperatures above 100 °C led to a significant reduction in glucosinolate content. In addition, the mirosinase enzyme can be inactivated at approximately 150 °C [46]. Moreover, glucosinolates may be lost through leaching into blanching water or volatilization during heat treatment [45,47].

On the other hand, mild heat treatments at temperatures 60–70 °C have little impact on glucosinolate. At these temperatures, myrosinase remains active, while epithiospecifier proteins (ESP), which convert glucosinolates into nitrile products, are inactivated. This allows for the preservation of beneficial isothiocyanates while preventing the formation of nitrile by-products [45,48]. In our study, the roasting method was performed at temperatures below 60 °C, which showed the highest ability to preserve glucosinolate content.

The antioxidant activities of Betong watercress extracts were demonstrated through their ability to reduce ferric complex compounds and scavenge DPPH and ABTS radicals. The efficacy of these activities varied depending on the drying method used. The roasting method showed the highest free radical scavenging ability, followed by the combination of roasting and drying, and finally the pretreatment method combined with the main drying method.

To assess the anti-diabetic property of Betong watercress, we focused on the pancreatic α-amylase enzyme inhibition. Pancreatic α-amylase is an enzyme that converts starch into polysaccharides, which are then broken down into glucose and absorbed into the bloodstream. Inhibiting α-amylase can help reduce blood glucose levels and shows potential for diabetes management [49]. In our study, the roasting method exhibited the greatest anti-diabetic ability, consistent with its superior antioxidant activity.

The study by Kittibunchakul et al. [37], as mentioned earlier, assessed the antioxidant and anti-diabetic properties of Sacha inchi seeds as well. The results showed that all three cooking methods, namely, roasting in a cooking pan, drying in a vacuum oven, and hot air-drying, significantly increased the FRAP value (4.27, 3.68, and 4.07 µmol TE/100 g dried weight) compared to the raw sample (2.47 µmol TE/100 g dried weight). Similarly, DPPH radical scavenging values showed that the roasting method (0.015 µmol TE/100 g dried weight) significantly enhanced antioxidant activity compared to the raw sample (0.012 µmol TE/100 g dried weight). For the α-amylase inhibition, the raw sample, roasted plant, and hot-air-dried plant showed no activity. However, Sacha inchi seeds dried in a vacuum oven at 0.4 mg/mL exhibited 16.33% inhibition of α-amylase. These results align with our findings, suggesting that roasting is the most effective method for enhancing antioxidant activity. During roasting, direct conductive heat transfer promotes the formation of Maillard reaction-derived antioxidants more effectively than cooking methods involving convective heat transfer, such as drying or boiling.

Our previous research further established strong correlations between polyphenol content (total phenolic and flavonoid content) and the antioxidant activities of Betong watercress, including FRAP, DPPH radical scavenging, ABTS radical scavenging, DPPH IC50, and ABTS IC50 values [50]. The structure of polyphenols, with an aromatic ring and hydroxyl groups, allows them to neutralize free radicals by donating hydrogen atoms from their hydroxyl groups. Moreover, molecules with hydroxyl groups, such as phenolic compounds, also exhibit potent inhibition of α-glucosidase and α-amylase enzymes, key enzymes in carbohydrate digestion [51]. 

Glucosinolate compounds and their secondary metabolites also play a role in glycemic control. Alderhami [52] studied cruciferous vegetables, such as *Carrichtera annua* L. (DC) and *Farsetia aegyptia* Turra. The results proved that glucosinolate in cruciferous plants, such as 4-methylthio-3-butenylglucosinolate, had a high ability to bind and inactivate pancreatic enzymes, including α-glucosidase and α-amylase. Furthermore, they revealed the good pharmacokinetic characteristics and lack of carcinogenic effects of glucosinolates. A review by Dedvisitsakul and Watla-Iad [53] further highlighted a positive correlation between antioxidant and anti-diabetic activities in plants. Therefore, the antioxidant and anti-diabetic potential of Betong watercress observed in this study may be attributed to its polyphenol content, glucosinolate content, and the conductive heat transfer during roasting.

The findings of this study demonstrate that Betong watercress, particularly when processed using roasting, retains a high concentration of bioactive compounds, including polyphenols, glucosinolates, and antioxidants. These compounds have shown significant potential for antioxidant and anti-diabetic activities, making Betong watercress a valuable functional food ingredient. Due to its rich phytochemical profile, it could be utilized in the development of natural supplements and functional food products aimed at preventing oxidative stress and managing diabetes. Furthermore, the use of roasting as a low-temperature drying method may enhance the nutritional value of cruciferous vegetables, making it a promising technique for the food processing industry.

Despite the promising outcomes, this study has certain limitations. First, we focused on the drying technique and did not explore variations in temperature and time during processing. These factors could have a significant impact on the preservation of bioactive compounds. Additionally, our study concentrated on the overall phytochemical content without identifying or quantifying individual phenolic acids, flavonoids, or glucosinolates. Moreover, this study relied primarily on in vitro assays, which do not fully represent the bioavailability or metabolic effects of these compounds in living organisms such as animals or humans.

Future research should aim to address these limitations and expand the current knowledge. Investigating the effects of varying temperature and time during the roasting process could provide deeper insights for industrial applications. Furthermore, it would be valuable to isolate and quantify individual phenolic acids, flavonoids, and glucosinolates to better understand their specific contributions to the bioactivity of Betong watercress. In vivo studies, including animal models and human clinical trials, are also necessary to confirm the bioavailability, metabolism, and efficacy of the antioxidant and anti-diabetic properties observed in vitro.

## 5. Conclusions

In summary, Betong watercress exhibits a high content of secondary metabolites, particularly polyphenols and glucosinolates, which contribute to its significant antioxidant and anti-diabetic activities. Among the drying methods investigated, roasting was identified as the most effective for preserving and enhancing the polyphenolic and glucosinolate content, thereby maximizing the antioxidant and anti-diabetic properties of Betong watercress. In contrast, combining drying methods with pretreatment, especially blanching, was found to reduce the levels of these beneficial compounds. These findings highlight the potential health benefits of Betong watercress and suggest that specific drying methods, such as roasting, are optimal for preserving its bioactive properties. Further research is needed to clarify the detailed mechanisms underlying these bioactivities and to optimize processing techniques for maximum health benefits.

## Figures and Tables

**Table 1:** Total phenolic content and total flavonoid content of Betong watercress with different drying processes.

Drying Processes	Total Phenolic Content ^1,2^	Total Flavonoid Content ^1,3^
R	36.27 ± 2.99 ^a^	6.58 ± 0.65 ^a^
RD	28.72 ± 1.21 ^b^	5.57 ± 0.08 ^ab^
BRD	24.46 ± 1.56 ^b^	4.38 ± 0.60 ^b^

^1^ Data are mean ± SD of triplicate determinations. ^2^ Total phenolic content is expressed as mg gallic acid equivalent (GAE) per gram of extract. ^3^ Total flavonoid content is expressed as mg quercetin equivalent (QE) per gram of extract. ^a,b^ Values in the same column following the different letter are significantly different (*p* < 0.05). R, roasting; RD, roasting and drying; BRD, blanching, roasting, and drying.

## Data Availability

The data presented in this study are available on request.
